# High Spatial Resolution Ensemble Species Distribution Modeling of *Rhodnius prolixus*, Vector of Chagas Disease, in Western Venezuela

**DOI:** 10.1029/2025GH001628

**Published:** 2026-05-20

**Authors:** Yan Gao, Nestor Añez, Luis Fernando Chaves

**Affiliations:** ^1^ School of Environmental and Geographical Sciences Shanghai Normal University Shanghai China; ^2^ Investigaciones Parasitológicas “J. F. Torrealba” Departamento de Biología Facultad de Ciencias Universidad de Los Andes Mérida Venezuela; ^3^ Department of Environmental and Occupational Health School of Public Health‐Bloomington Bloomington IN USA; ^4^ Department of Geography Indiana University Bloomington IN USA

**Keywords:** MODIS, machine learning, *Trypanosoma cruzi*, population at risk, remote sensing

## Abstract

*Rhodnius prolixus* is the most common and abundant kissing bug found in Royal and other native palms from western Venezuela. *R*. *prolixus* is a dominant vector of *Trypanosoma cruzi*, the parasite causing Chagas disease. Here we use species distribution models (SDMs) to estimate habitat suitability for *R*. *prolixus*. Based on habitat suitability we estimate the population at risk of Chagas disease transmission. We fitted an ensemble SDM with 250 m spatial resolution using remote sensing covariates, processed for the same time when kissing bugs were sampled, for modeling *R*. *prolixus* habitat suitability, based on 67 samples (from 41 locations) collected between 2004 and 2012. The ensemble SDM included prediction using six different machine learning algorithms, which include: generalized linear model, multiple adaptive regression splines, regression trees, random forests, generalized boosted regression trees, and extreme gradient boosting. The final SDM included 9 out of 13 variables selected using variable importance. The final ensemble SDM had an average receiver operating curve (±SD) of 0.833 ± 0.114 for the best model. The model suggested a high habitat suitability for *R*. *prolixus* along the eastern slope of the Venezuelan Andean Cordillera spread along the states of Merida, Barinas, Tachira, Trujillo, and Portuguesa. Validation with an independent data set, collected between 2012 and 2018, showed that higher suitability predicted occurrence of *R*. *prolixus* (*p* < 0.025). The results show how ensemble SDMs can provide high spatial resolution distribution information for *R*. *prolixus*, which can be used to accurately estimate Chagas disease transmission risk.

## Plain Language Summary

*Rhodnius prolixus* is a kissing bug that transmits parasites causing Chagas disease. Using tools to map its distribution can help to estimate how many people live near kissing bugs and might be at risk of contracting Chagas disease. Here, we use a model to illustrate the preferred environmental conditions this kissing bug prospers in. We found that this kissing bug lives at low elevations, in a warm environment with a temperature that does not change much, while preferred vegetation conditions, can vary from very sparse to very dense. When we mapped the model, we found this kissing bug was very common along the eastern slope of the Venezuelan Andean Cordillera. Based on the area where this kissing bug is likely present we estimated the population at risk of contracting Chagas disease. Current estimates range from 82,296 to 1,943,934 people at risk of Chagas disease for the studied area in 2020.

## Key Points

- An ensemble species distribution model was used to estimate habitat suitability for *Rhodnius prolixus* in western Venezuela
- High habitat suitability for *R. prolixus* was found along the eastern slope for the Venezuelan Andean Cordillera
- Population exposed to *R. Prolixus* decreased as habitat suitability threshold increased (0.6–0.9), but increased through time (2000–2020)

## 1 Introduction

Species distribution models (SDM) address questions about where species live, what environmental factors determine the presence (or occurrence) of a species across a landscape, and unsampled areas where conditions for the species occurrence might exist (Elith & Leathwick, 2009). SDMs are powerful computational tools that bridge the gap between species occurrence data, environmental conditions and the potential distribution of the species in a region. SDM function by using statistical techniques and/or machine learning algorithms to analyze the relationship between known locations where a species has been observed (or recorded as absent) and the environmental characteristics of those locations, such as climate (temperature, precipitation), topography (elevation, slope), soil properties, land cover, or proximity to water (Miller, 2010). Essentially, SDMs create digital maps of habitat suitability, that is, the probability that a species might be present across locations in a landscape (Rhodes et al., 2022).

Here, we focus on using SDM to predict the spatial distribution of Chagas disease vector *R. prolixus*. In SDM, species occurrence data are species locations typically from field research. Environmental predictors often refer to raster layers of remote sensing and climate data sources such as land surface temperature (LST) from MODIS (Wan et al., 2015), vegetation indices including normalized difference vegetation index (NDVI) and enhanced vegetation index (EVI) (Rouse et al., 1973), and climate data from WorldClim (Hijmans et al., 2005). Model algorithms include statistical and machine learning algorithms which allow finding the relation between species location and environmental variable (Boehmke & Greenwell, 2020).

*R. prolixus* is a triatomine species with an ample geographical distribution in northern south American countries including Colombia, Venezuela, Guyana, Suriname, French Guiana (an Overseas Department of France), and the amazon basin of Brazil, being Venezuela, and Colombia the countries where this kissing bug species is the most abundant and the most important vector of *Trypanosoma cruzi* (Gorla & Noireau, 2010; Gurgel‐Goncalves et al., 2012). The spatial distribution of *R. prolixus* has been modeled using environmental predictors derived from coarse grain resolution (8 km) remote sensing data, including climate, topography, land cover and use and spectral measurements (Ceccarelli et al., 2015). Artificial intelligence techniques including machine learning algorithms have been applied to predicting models for the spatial distribution of insect vector species (Rhodes et al., 2022). Chagas disease vector distribution has been modeled using an ensemble approach averaging suitability across different machine learning algorithms, including MaxEnt, random forest (RF), support vector machine, and Bayesian Gaussian models (Brasil et al., 2025). However, this approach has not been applied to specific dominant vector species like *R. prolixus*.

In Venezuela, *R. prolixus* is common in landscapes from 4 to 2,000 m and constitutes about 90% of all triatomines collected in intradomicile or peridomicile environments. This kissing bug species is associated with several mammal wildlife species including: *Didelphis marsupialis*, *D. albiventris*, *Agouti paca*, *Dasypus novemcinctus*, *Coendou prehensilis*, as well as *Attalea* spp., *Acrocomia* spp., *Copernicia* spp. palms, reptiles, and bird‐nests (Añez et al., 2017, 2021; Gamboa, 1963; Gómez Núñez, 1963; Lent & Wygodzinsky, 1979; Rabinovich et al., 1979).

The importance of *R. prolixus* for human health was first suggested by Brumpt (1913) after obtaining an experimental infection with *T. cruzi* in nymphs and adults of this kissing bug species sent to him from Venezuela. Later, Tejera (1919) working in a rural village located in the Maracaibo Lake basin, in western Venezuela, found several specimens of *R. prolixus* naturally infected by Trypanosomas morphologically identical with *T. cruzi*. The inoculation of these protozoa on small rodents led to the observation of protozoa similar to those observed on blood samples from human patients from the same region where kissing bugs were collected. Similarly, Uribe (1922) during an investigation carried out in the laboratory of the Venezuela Sun Oil Company in Valera‐Venezuela, reported that *R. prolixus* was widely distributed in Trujillo state, part of the Andean region of Venezuela, including places at 1,300 m. Uribe (1922) also described *R. prolixus* as strictly domiciliary, that is, living inside rural houses. However, Gamboa (1963) demonstrated, for the first time in Venezuela, the presence of all *R. prolixus* developmental stages in palm trees, and also in nests of herons and other birds commonly found in the Llanos region of Venezuela, which is east of the Venezuelan Andes. The study by Gamboa (1963) expanded the knowledge on the ecology of *R. prolixus*. This foundational study also contributed to a better interpretation of Chagas disease from the eco‐epidemiological point of view. More recently, Añez et al. (2017) studied kissing bug samples from 95 palms in 41 localities from five states in western Venezuela. This study found 96% of the kissing bugs were *R. prolixus*. Añez et al. (2021) reported the presence of adult *R. prolixus* in houses located in rural and urban areas of the Andean region, whose elevation ranged from 20 to 895 m.

*R. prolixus* bloodfeeds on a multitude of mammal reservoir species, leading to complex sylvatic, peri‐domestic, and domestic *T. cruzi* transmission cycles. In certain areas, the maintenance of the domestic cycle by sylvatic kissing bugs makes vectorial transmission control a difficult task. During recent decades the Venezuelan Chagas disease control program has weakened, and vectorial transmission hasn't been interrupted but instead increased (Feliciangeli et al., 2003). Moreover, recent observations of acute cases in absence of intradomicile vector populations in Barinas State (Añez et al., 1999; Feliciangeli, Sánchez‐Martín, et al., 2007) and Mérida State (Feliciangeli et al., 2002) suggest new eco‐epidemiological transmission scenarios.

Prior SDMs for *R. prolixus* in Venezuela have been performed at relatively coarsely grained spatial scales between 1 and 8 km (Bender et al., 2020; Ceccarelli & Rabinovich, 2015; Ceccarelli et al., 2015; Eduardo et al., 2021), and these efforts have mostly relied on the use of single algorithms, such as MaxEnt (Altamiranda‐Saavedra et al., 2024), which have not been evaluated as part of ensembles, known to increase the precision and accuracy of SDMs (Brasil et al., 2025).

Several ecological factors could be considered important for shaping *R. prolixus* distribution in western Venezuela. These include the presence of palms (i.e., *Attalea* spp., *Acrocomia* spp., *Copernicia* spp.); the climate presented by a temperature range of 25°C–35°C, relative humidity of 75%–80% and the presence of blood sources from vertebrate animals. In light of these observations, our hypothesis is that the distribution of *R. prolixus* is associated with environmental factors such as temperature, elevation and vegetation, all of these variables that can be measured with remote sensors in satellites, and that can also be used to produce habitat suitability estimates based on SDMs. From the perspective of vector control operations, SDMs at 250 m resolution vector maps can also improve current methodologies to estimate the size of human populations exposed to kissing bugs, or the entomological risk of Chagas disease transmission, which currently is based on qualitative ranges for dominant vector species (PAHO, 2025).

Our objective is to create an ensemble SDM for *R. prolixus* and to use the resulting model to estimate the population at risk of Chagas disease in the studied area of western Venezuela. We use remote sensing data (250 m resolution) and the following algorithms: generalized linear model (GLM), multiple adaptive regression splines (MARS), regression tree (RT), RF, generalized boosted regression trees (GBM), and eXtreme gradient boosting (XGBOOST) to build an ensemble SDM for *R. prolixus*. We validate the resulting habitat suitability map based on predictions from the ensemble SDM with an independent data set of kissing bug occurrences and absences not used in the ensemble SDM estimation. Finally, we illustrate how habitat suitability maps from the ensemble SDMs of insect vectors can be used to estimate the entomological transmission risk of a vector‐borne disease.

## 2 Study Area and Data

### 2.1 Study Area

**Figure 1:**
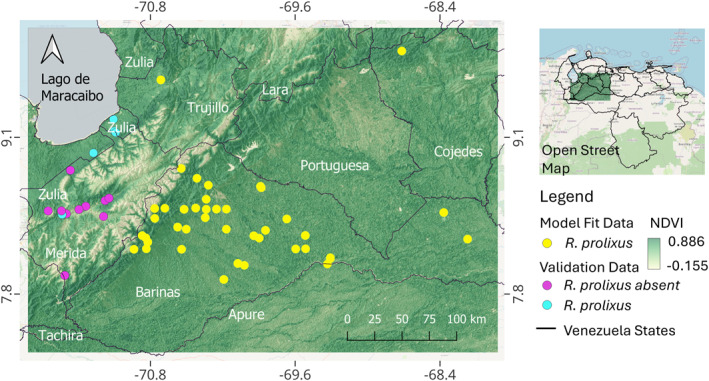
The study area in western Venezuela presented using a normalized difference vegetation index image over a hillshade product generated by employing a digital elevation model. The elevation ranges from 0 to 4,750 m. The yellow points represent 41 *Rhodnius prolixus* occurrence locations which were used to fit the *R. prolixus* ensemble species distribution model. Kissing bugs were collected during 2004–2012 (Añez et al., 2017). The cyan points represent five known *R. prolixus* occurrences and the purple points 10 absences, collected during 2012–2018 (Añez et al., 2021). These locations were used to validate the final ensemble species distribution models.

The study area, where *R. prolixus* sample data was collected, comprises five states of western Venezuela, Barinas, Apure, Cojedes, Trujillo, and Merida, the first three belong to the Llanos region, and Trujillo, and Merida to the Andean foothills west of the Llanos region (Figure 1). The elevation of the study area ranges from 0 to 4,980 m, and the temperature varies from 10 to 32 degrees Celsius (°C). The relative humidity is above 75% and the mean rainfall is over 1,500 mm/year, and the area is characterized by a dry season, from September to March, and a rainy season, from April to August (Chaves & Vivas, 1972). Despite the wide range of elevation in the study area, kissing bugs have only been found below 2000 m of elevation, with a warm temperature between 28°C and 32°C.

### 2.2 Entomological Data

The species distribution model (SDMs) was based on 95 sampled palms with the presence of *R. prolixus* collected during 2004–2012. The 95 palm locations were reduced to 41 locations when considering palm clusters where palm to palm distance was below 250 m. For the clusters we used the location of the most central palm. The collection and processing of the samples were described in detail by Añez et al. (2017). Fifteen extra palms were sampled during 2012–2018 with five *R. prolixus* occurrences and 10 known absences. These 15 additional locations were used for ensemble SDM validation (Añez et al., 2021). The location of the sampled points is presented in Figure 1.

### 2.3 Remote Sensing Data

**Figure 2:**
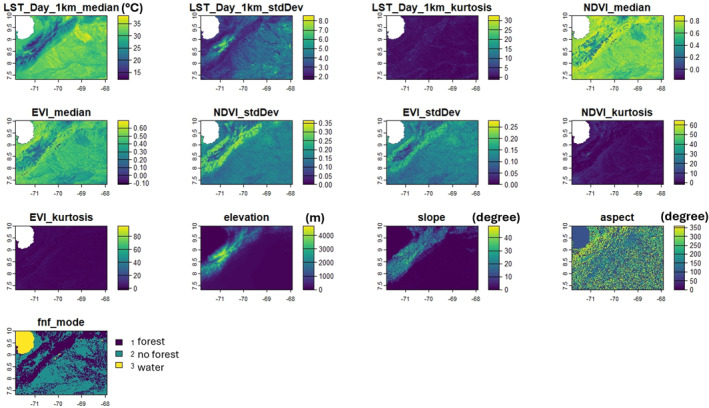
The 13 layers of covariates for the species distribution model for *R. prolixus*. Each covariate is indicated above each panel. Bars to the right of each panel are scales for the covariates. Land surface temperature is in degree Celsius (°C), elevation in meters (m), slope and aspect are measured in degrees. For the phased arrayed L‐band synthetic aperture radar forest no forest classification (fnf_mode), 1 represents forest, 2 no forest, and 3 water. Normalized difference vegetation index and enhanced vegetation index are dimensionless indices.

The covariates for the SDMs included LST, vegetation indices, topographic data for aspect, slope, elevation, and a forest no forest land use land cover classification (Figure 2). We downloaded composite images for the median of images from the 2004–2012 period from the Google Earth Engine (GEE) platform using JavaScript code available on the GEE website (Google Earth Engine, 2020; Gorelick et al., 2017). For the estimation of composite images, we employed daily images, from 1 January to 31 December of each considered year, for LST with a spatial resolution of 1 km (MOD1101, Version 6) based on Moderate Resolution Imaging Spectroradiometer (MODIS) images (Wan et al., 2015). These images were re‐scaled by multiplying each pixel by a factor of 0.02, and the resulting temperatures were transformed from °K to °C by subtracting 273 (Chaves et al., 2021). We employed MODIS NDVI and EVI available at 250 m spatial resolution, both of which are considered proxies for vegetation growth and sampled every 16 days (Didan, 2015). Each image in the resulting LST and vegetation index time series had the cloud cover removed before the estimation of the composite images (Chaves et al., 2021). We also included a Digital Elevation Model (DEM) from the NASADEM_HGT v001 data at 30 m spatial resolution (NASA JPL, 2020). We estimated slope and aspect from the DEM following the methodology described by Rhodes et al. (2022). Slope and aspect were measured in degrees. Slope is the rate of elevational change of the landscape measured in the steepest direction at any point, while aspect is the direction in which the slope is measured (where 0 is north, 90 is east, 180 is south, and 270 is west). The forest no forest land use land cover classification was based on advanced land observing satellite phased arrayed L‐band synthetic aperture radar (PALSAR) images (Shimada et al., 2014). The PALSAR forest classification is based on identified forests with an area larger than 0.5 ha, with over 10% forest coverage in accordance with the Food and Agriculture Organization forest definition (Shimada et al., 2014). The PALSAR yearly product was at 25 m spatial resolution, with three categories, where one, two, and three represent forest, no forest, and water, respectively. For PALSAR we estimated the mode of the annual images available for 2004–2012. All the data layers were projected at WGS 84 (EPSG 4326) geographic coordinate system and were reprojected to a 250 m spatial resolution.

With the GEE we also estimated composite images for the standard deviation (SD) and Kurtosis MODIS LST, MODIS NDVI, and EVI images time series. We incorporated statistical moments of SD and Kurtosis with the intention to test the possibility that *R. prolixus* is sensitive to patterns of variability in the environment, as predicted by Schmalhausen's law, the biological principle stating that organisms follow both the mean and higher order moments, that is SD and kurtosis, of environmental variation (Chaves, 2017; Chaves et al., 2012).

Since we only had occurrences of *R. prolixus*, we needed to simulate pseudoabsences, that is, we generated random locations where *R. prolixus* might be absent. We generated pseudoabsences using the species range envelope (SRE) method. SRE defines a species' potential habitat by constraining the range of environmental variables where a species could be found according to presence data. This process creates an “envelope” which is used to generate pseudoabsences by choosing points outside the SRE. In this method, points for locations where *R. prolixus* could have been absent were randomly sampled from areas considered environmentally dissimilar from the locations of the observed occurrences (Barbet‐Massin et al., 2012).

## 3 Methods

### 3.1 Variable Selection

Given the relatively large number of covariates, and potential issues with inference when variables are collinear or repetitive, we used model selective to eliminate variables. We specifically employed variable importance for model selection. Variable importance is estimated by randomly shuffling variables via permutation. If a variable does not explain a response, this permutation will have little effect when compared with a variable that explains more successfully a response, and this can be measured as the percentage of times the model, with the original variable, outperforms the model with the shuffled variable. In our models we estimated variable importance with 100 permutations and removed all variables whose importance was below 5% (Rhodes et al., 2022, 2023).

### 3.2 Model Evaluation

We evaluated the models using three different metrics, including: Kappa coefficient, the area under the receiving operating curve (ROC) and the true skill statistic (TSS) (Allouche et al., 2006). The Kappa coefficient measures the agreement between observations and predictions, with values ranging from 1, when perfect agreement occurs, to −1 for a total disagreement. The ROC statistics estimate the separation between occurrences and absences, with a value of 0.5 suggesting a prediction no better than random chance. The TSS statistics is the sum of model sensitivity and specificity minus one (Guisan et al., 2017).

### 3.3 Ensemble Species Distribution Model

To build the ensemble SDM, we employed the following classification methods: GLM, MARS, RT, RF, GBM, and XGBOOST. GLM is a classical statistical model using a linear function to model occurrences and absences (Rhodes et al., 2023). MARS relaxes linearity assumptions and allows non‐linear functions built using splines (Rhodes et al., 2022). The remaining methods are tree‐based machine learning algorithms. RT partitions the response variable recursively across the covariate space, forming branches that predict the response variable while minimizing the error (Kuhn & Johnson, 2013). RF is the equivalent of a non‐parametric bootstrap for trees, by which trees are built with new response variables from previous trees and integrating residuals sampled with replacement (Kuhn & Johnson, 2013). Finally, GBM is a method that combines simpler trees to improve predictions, based on the observation that averaging outcomes reduces errors in predictions (Bühlmann & Yu, 2010). GBM and XGBOOST differ mainly in how trees are boosted, where the later optimizes the process through optimization of gains as boosted trees are generated (Boehmke & Greenwell, 2020).

For the ensemble, a random strategy was used for cross validation that was repeated three times for the 10 data sets of pseudoabsences generated for *R. prolixus*, where 80% of the points (occurrences and pseudoabsences being equally weighted) were used for model fitting and the remaining 20% for model evaluation to compute the Kappa coefficient, ROC and TSS. A final ensemble model was fit to include all models with a TSS score of 0.7 or higher, where each of the selected model was weighted proportionally to its TSS score. We only consider TSS scores of 0.7 and above to ensure high model performance (Rhodes et al., 2022, 2023).

### 3.4 Model Validation

SDM validation was done using both occurrences and absences, from data collected between 2012 and 2018 described in Section 2.2. The hypothesis is that occurrences of *R. prolixus* will have a higher probability for habitat suitability than *R. prolixus* absences. We employed a one tailed Wilcoxon test to check whether the difference between both probability distributions (occurrence vs. absence) was significant (Hollander et al., 2013).

### 3.5 Model Uncertainty

Following Rhodes et al. (2023) we employed kurtosis to measure the agreement between different models conforming the ensemble SDM. Kurtosis is formally defined as the fourth standardized moment of a statistical distribution (Ross, 2014) and is a metric that differentiates if variability in model predictions from the ensemble is concentrated toward the mean of a distribution (a leptokurtic distribution associated with higher kurtosis values) or if model predictions differ considerably and are far from the mean prediction (a platykurtic distribution associated with a lower kurtosis value). This metric, unlike other suggestions such as the SD of the predictions (Woodman et al., 2019), can separate high uncertainty predictions (platykurtic) from low uncertainty predictions (leptokurtic) and is independent of the intrinsic variability of predictions that shape the unscaled variance of a distribution (Ross, 2014).

### 3.6 Estimation of Population at Risk of Chagas Disease Transmission

Assuming that the entomological risk of Chagas disease transmission is driven by kissing bug vector presence (PAHO, 2025), we used a series of five maps of human population counts, for the 2000–2020 period. These maps provide population estimates in steps of 5 years and are based on data from national censuses and population registers at 30 arc‐second (∼1 km at the equator) resolution (CIESIN, 2017). We used these population estimates to assess the entomological risk of acquiring Chagas disease based on habitat suitability estimates for *R. prolixus*. For this we generated vector maps of presumed *R. prolixus* presence based on habitat suitability thresholds of 0.6, 0.7, 0.8, and 0.9 using QGIS (3.28.12) and *R* (4.3.2), so that we could generate estimates that consider uncertainty in the probability of vectors being present in an area with a given number of people. Using the function “resample” from the *R* terra package, we resampled habitat suitability map (250 m) to the spatial grain of 1 km which is the native resolution of the human population count maps, given the suggestion of not reprojecting CIESIN maps. We exported the resampled habitat suitability map in 1 km spatial resolution to QGIS, and using command reclassify by table in raster analysis we generated binary maps of habitat suitability in which 1 represent areas with suitability above the previously mentioned threshold, and 0 below the threshold. We converted these binary maps to vector using command raster/conversion/polygonize (raster to vector). Using the function “zonal” from the *R* terra package, we then summed the values for the areas intersected by the *R. prolixus* habitat suitability maps and the population maps.

### 3.7 Implementation

All procedures described in this methods section were done using freely available software: QGIS version 3.28.2 (QGIS development team, 2024) and *R* version 4.3.2 (R Core Team, 2023). We employed a wide range of functions and packages. Reclassification and zonal statistics of raster data were done in QGIS using function of “reclassify by table” and “raster layer zonal statistics.” Spatial data processing and mapping were done using the WGS84 reference system via the *R* packages “terra” (Hijmans, 2025) and “tidyterra” (Hernangómez, 2023). Map color palettes were made using “paletteer” (Hvitfeldt, 2021). Ensemble SDM was performed using “biomod2” (Guéguen et al., 2025). Variable importance plotting was done using “gplots” (Warnes et al., 2024) and the Wilcoxon test was made using “coin” (Hothorn et al., 2006). SDM fitting in *R* was parallelized using 6 cores on an Intel(R) Core (TM) i5‐10310U CPU @ 1.70 GHz, with 8.0 GB of memory.

## 4 Results

**Figure 3:**
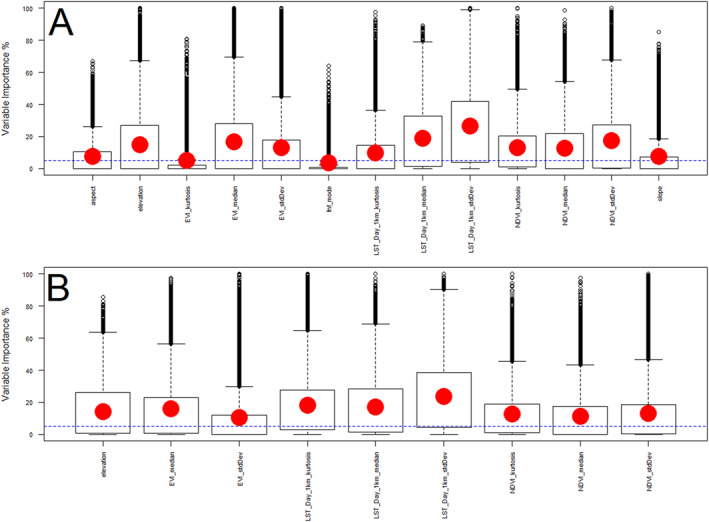
Covariate selection by variable importance for an ensemble species distribution model of *R. prolixus*. Panel (a) shows variable importance for the full 13 covariates described in the methods section. Panel (b) shows the nine covariates with the highest variable importance considered in the best model. In panels (a, b) boxplots show the distribution of variable importance, where the box shows the second and third quartiles of the distribution, large red dots the means, and black circles represent outliers. The best model, presented in panel (b), included nine variables from MODIS: the three statistical moments of MODIS land surface temperature (standard deviation [SD], kurtosis, and median), the median and SD of the enhanced vegetation index, and the three statistical moments of the normalized difference vegetation index (NDVI, SD, kurtosis, and median). The ninth variable was elevation from NASADEM.

From 13 environmental variables, we ended up with nine covariates after model selection, and model performance was improved when comparing the full model (13 covariates) with the best model (nine covariates) according to Kappa, ROC, and TSS (Table 1). Figure 3 shows variable importance boxplots for covariates modeling habitat suitability for *R. prolixus*.

**Table 1:** Predictive Ability of the Full and the Best Model Explaining Habitat Suitability for *R. prolixus*

Full (13 covariates)			Best (9 covariates)	
Metric	Average	SD	Average	SD
KAPPA	0.425	0.231	0.558	0.216
ROC	0.761	0.127	0.833	0.114
TSS	0.424	0.231	0.556	0.215

*Note.* Metrics include the kappa coefficient, the receiver operator curve (ROC), and the true skill statistic (TSS). We report both the average and standard deviation (SD) of the three metrics.

**Figure 4:**
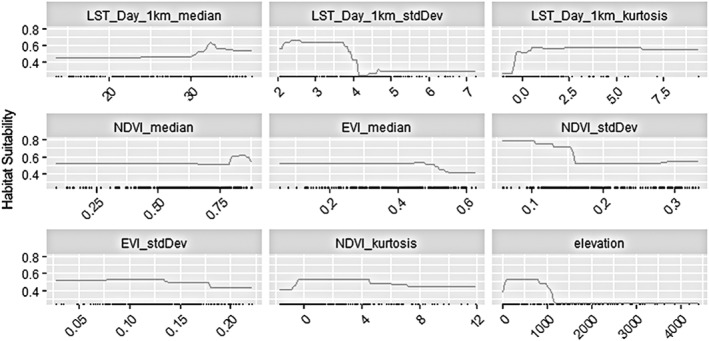
Strip plots of the predicted *R. prolixus* habitat suitability (probability of presence) as function of the covariates considered in the ensemble species distribution model of the best models. In the plots, each line represents the median for the replicated runs of each model whose TSS > 0.70. Each panel represents the following covariates (from left to right starting in the top column): land surface temperature (LST) median; LST standard deviation (SD); LST kurtosis; normalized difference vegetation index (NDVI) median; enhanced vegetation index (EVI) median; NDVI SD; EVI SD; NDVI kurtosis and Elevation.

The nine selected covariates included (Figure 4): LST median, which measures median LST, showed a nonlinear association, where temperature over 30°C was associated with a sharp increase in suitability, however the suitability reached a plateau around 32°C and decreased around 33°C, LST SD, which measures the SD of LST, the relationship with habitat suitability being highly nonlinear, with suitability decreasing sharply after about 3.5 units of SD and reaching the lowest suitability after 4.5 units of SD, showing *R. prolixus* is more likely to be present in environments with low variability in temperature. LST kurtosis suggested temperature distributions concentrated toward the mean (high kurtosis or leptokurtic, i.e., concentrated near the mean with low variability) increased habitat suitability. Vegetation cover and its variability were also associated with habitat suitability of *R. prolixus*. NDVI above 0.75 was associated with the highest habitat suitability estimates. EVI around 0.5 maximized habitat suitability, and values outside that range were associated with lower suitability suggesting *R. prolixus* is present where vegetation is neither too low nor too high according to EVI, but relatively high for NDVI. Certain variability in vegetation density is favorable for *R. prolixus*, reflected in NDVI and EVI SD, as well as in NDVI kurtosis. Elevation between 50 and 1,000 m are associated with high suitability, and suitability decreases after 1,000 m and reaches its lowest at about 2,000 m, suggesting that *R. prolixus* is unlikely present at high elevations.

**Figure 5:**
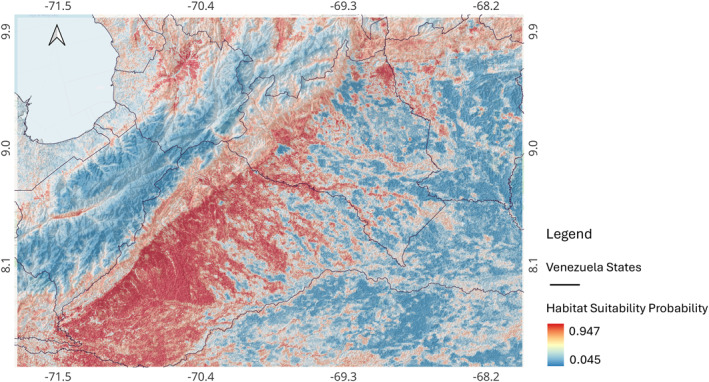
Ensemble species distribution model for *R. prolixus* habitat suitability in western Venezuela. Color indicates habitat suitability measured as a probability (0–1), higher probability indicates higher suitability. In the map the *Y* axis is the latitude, and the *X* axis is the longitude.

The area with the highest suitability for *R. prolixus* was spread across the states of Barinas, Tachira, and Portuguesa, mainly along the eastern slope of the Venezuelan Andean Cordillera (Figure 5). While in other states of the study area, Trujillo, Merida, Lara, Portuguesa, Zulia, and Cojedes, the presence of high suitability is spatially disrupted. The average habitat suitability for the study area (average ±SD) was 0.45 ± 0.28.

**Figure 6:**
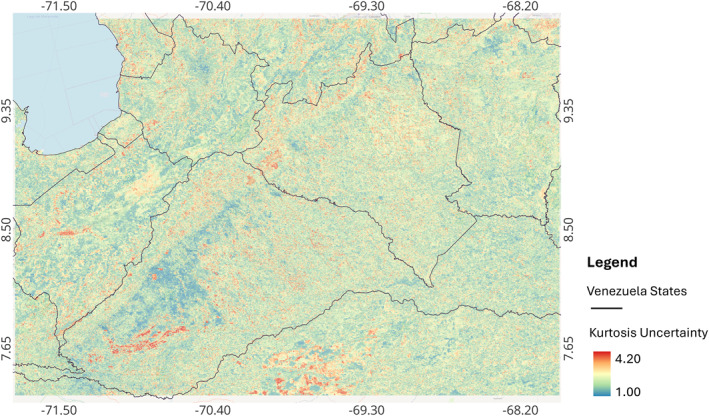
Uncertainty for *R. prolixus* habitat suitability based on an ensemble species distribution model (species distribution models [SDM]) for western Venezuela. Color indicates kurtosis in the habitat suitability from the models employed to ensemble SDM, higher kurtosis values indicate less uncertainty. In the map the *Y* axis is the latitude, and the *X* axis is the longitude.

The uncertainty of the ensemble SDM is presented in Figure 6. The uncertainty is highly granular, suggesting that the areas at elevations where the Andean piedmont starts (around 1,000 m) are the ones with the highest uncertainty, which then decreases as elevation decreases.

**Figure 7:**
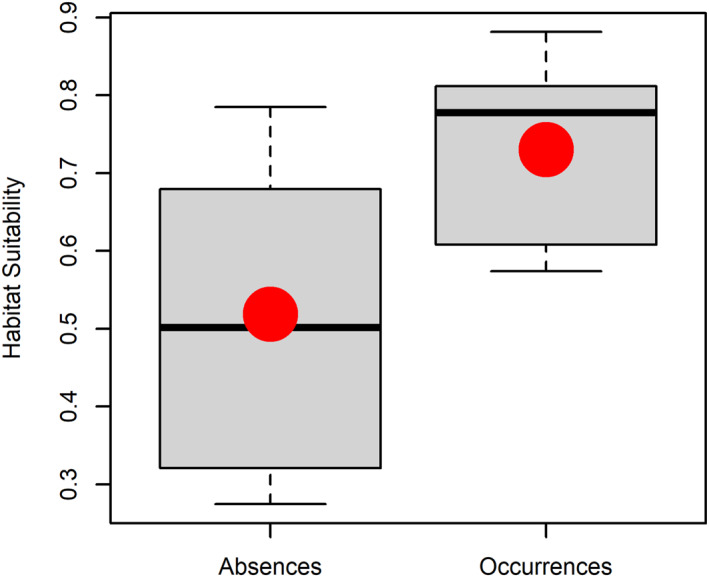
Boxplots showing the distribution of absences and occurrences of *Rhodnius prolixus* in native palms from Merida, Venezuela collected between 2012 and 2018. The mean value (±SD) of absences was 0.519 ± 0.193 (*n* = 10) and of presences was 0.731 ± 0.133 (*n* = 5). In the boxplots, the box represents the second and third quartiles of the observations and horizontal black lines inside the boxes are the medians of the distribution, the red dots indicate the mean values of the distribution.

We validated the *R. prolixus* habitat suitability ensemble model using the locations of five occurrences and 10 absences. We compared the distribution of the suitability for occurrences and absences (Figure 7) using a one tail Wilcoxon test, where the alternative hypothesis is that probability of occurrences is higher than absences (Hollander et al., 2013). We found that the average suitability for occurrence sites was significantly higher than for absent sites (*z* = −1.96, *p*‐value = 0.025). This result validates the ensemble SDM, which was built using samples collected across what our results suggest is a critical elevational gradient for the presence of *R. prolixus*.

**Figure 8:**
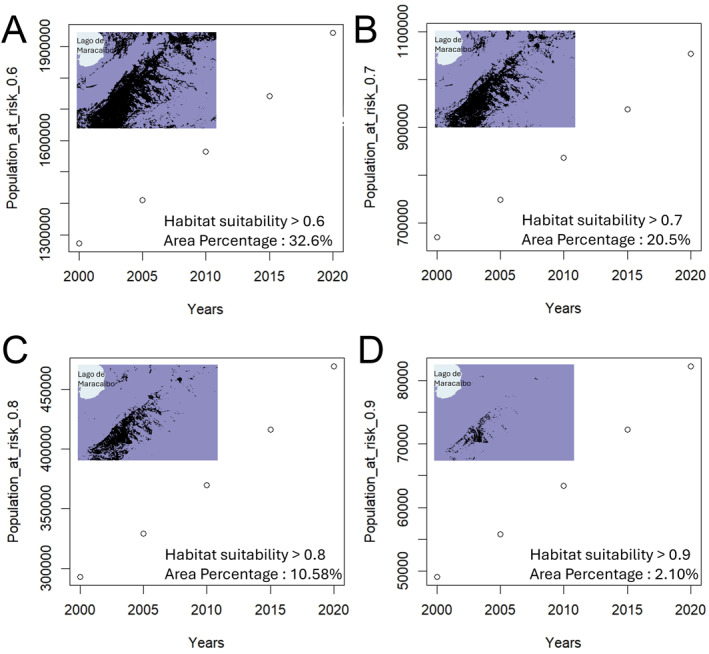
Population at risk of Chagas disease transmission by *Rhodnius prolixus* with a habitat suitability threshold of 0.6 (a), 0.7 (b), 0.8 (c), and 0.9 (d).

The estimates of population with an entomological risk for Chagas disease transmission are presented in Figure 8. In that figure each panel shows the estimated range for *R. prolixus* and the estimated population with an entomological risk of transmission (or entomological exposure) every 5 years between 2000 and 2020.

In general, we observed that while the population at risk decreased when increasing the habitat Suitability threshold, the population at risk of Chagas disease increased through time. For example, in 2000, estimates of population at risk ranged from close to 49,050 (0.9 suitability threshold) to 1,272,342 (0.6 suitability threshold), while in 2020, such estimates of population at risk increased to 82,296 (0.9 suitability threshold) and 1,943,934 (0.6 suitability threshold).

## 5 Discussion

This study mapped the habitat suitability for *R. prolixus* in western Venezuela using high quality entomological data and environmental data collected with remote sensors and analyzed using an ensemble species distribution model (SDM) that combined predictions from six machine learning algorithms. The results showed high suitability for *R. prolixus* along the eastern slope of the Venezuelan Andean Cordillera, in the states of Barinas, Tachira, and Portuguesa. While in the rest of the states, such as Trujillo, Merida, Lara, and Cojedes, the mapped suitability area is more fragmented (Figure 5). Our finely grained spatial predictions (0.0625 km^2^) for *R. prolixus* habitat suitability in western Venezuela align well with previous studies where the occurrence of *R. prolixus* was predicted, by models with a coarser spatial resolution between 5 and 25 km^2^, to be highest in northern Venezuela (Bender et al., 2020) and also in the piedmont next to the Andes Cordillera (Calderón & González, 2019), the area with the highest predicted habitat suitability in our ensemble model.

The prediction of habitat suitability for *R. prolixus* was based on nine variables, most of which had sigmoid forms, meaning that suitability either went up or down around some critical threshold value. Critical values in LST, NDVI, EVI and elevation can inform us of the suitable environmental conditions in temperature and vegetation that *R. prolixus* is likely to be present. The optimal LST for *R. prolixus* was found at 32°C–33°C retrieved from satellite‐based MODIS LST product. Previous work has shown that *R. prolixus* thrives in temperatures between 28°C and 30°C measured at 2 m above ground (Añez et al., 2017). Although MODIS LST has been identified as having the highest agreement with surface air temperature (AT) (Urban et al., 2013), there are discrepancies between LST and near surface AT. The comparisons between LST and AT vary by regions and seasons (Vancutsem et al., 2010). MODIS LST tends to overestimate surface temperature during daytime, while underestimate surface temperature during nighttime (Mildrexler et al., 2011). The discrepancy varies also with land cover types. For example, in barren land, the discrepancy tends to be larger while in forest areas the discrepancy diminishes (Mildrexler et al., 2011). However, for the purposes of this study LST seems to be an appropriate choice to consider the impacts of temperature on habitat suitability for *R. prolixus*, yet the values shouldn't be taken at face value given these predictions are based on remotely sensed data that has not been compared with data from in situ sensors, which will ultimately provide more robustness to model predictions and will allow the interpolation about environmental conditions experienced by kissing bugs.

Because *R. prolixus* was sensitive to the higher order statistical moments of covariates, for example, for both LST and NDVI, SD and kurtosis, and for EVI, SD, *R. prolixus* is sensitive not just to the median but also to the full variability pattern of environmental covariates. This includes how much variability is in the environmental covariates (measured by SD), or whether the distribution is concentrated toward the mean or to the extremes of the covariate distribution (measured by Kurtosis). This type of behavior is predicted by Schmalhausen's law (Chaves, 2017; Chaves & Koenraadt, 2010; Chaves et al., 2012; Lewontin & Levins, 2000), the biological principle stating that organisms are sensitive not only to average environments, but also to their patterns of variability. The fact that LST SD was the most important covariate, followed by LST Kurtosis and LST median shows that the patterns of variability in LST (how much LST varies) are more important for *R. prolixus* suitability prediction than LST in average. NDVI related covariates show a similar pattern, suggesting that the pattern of variability in NDVI (how much NDVI varies around its mean value) is more important for *R. prolixus* suitability prediction than average NDVI conditions. It is worth noting that LST kurtosis (which was leptokurtic) and NDVI Kurtosis (which was platykurtic, i.e., with wide variability around the mean) had opposite impacts on *R. prolixus* habitat suitability. This indicates that *R. prolixus* prefers a narrow range of LST. In this range *R. prolixus* can be present across a wide range of vegetation conditions, from very sparse, degraded vegetation to dense, highly preserved vegetation.

Elevation, as a topographic variable, is an important covariate for habitat suitability for *R. prolixus*. The range of elevation corresponding to the area with high suitability (probability >0.8 and 0.9) in our predicted map is from 324 to 1,029 m (with habitat suitability increasing as elevation decreases), which is within the previous study 0–2,000 m in elevation of observed habitats for *R. prolixus* (Añez et al., 2017), and makes essential the surveillance of this kissing bug across the altitudinal range of the Andes cordillera, as the species might expand into higher altitudes with global warming, as observed for several insects and, more generally, for ectothermic organisms (Chaves, 2016, 2017; Chaves & Friberg, 2021; Parra‐Henao et al., 2016; Ramalho et al., 2023).

Mapping habitat suitability for *R. prolixus* continues to be an important topic because of kissing bugs' ability to efficiently transmit *T. cruzi*. This vector occurs in high abundance in palms, where it is associated with reservoirs that perpetuate transmission cycles (Gómez Núñez, 1963), and it also colonizes rural domiciles with mud walls and thatched roofs (Rabinovich et al., 1979) which are habitats common among the poorest of the poor in rural Venezuela (Briceño‐León, 1987). Moreover, *R. prolixus* has an extraordinary ability to infest and re‐infest domiciles from nearby natural habitats (Feliciangeli, Sanchez‐Martin, et al., 2007; Fitzpatrick et al., 2008; Sanchez‐Martin et al., 2006), a short time from bloodfeeding to defecation (Killets et al., 2025; Kirk & Schofield, 1987; Pippin, 1970) and a high *T. cruzi* infection prevalence in the field (Añez et al., 2017, 2021; Feliciangeli, Sánchez‐Martín, et al., 2007; Gamboa, 1963). These traits that ease *T. cruzi* transmission pose an increased risk to people that are close to its presence, especially to those that live under socioeconomically disadvantaged conditions (Briceño‐León, 1990). The SDM, and current knowledge about the biology of kissing bugs, also suggest that with current changing environmental patterns the distribution of *R. prolixus* will change. For example, Tamayo et al. (2018) found that the expected temperature increase of 2°C under climate change could cause an increase in *R. prolixus* abundance and a greater probability of infection by *T. cruzi*, which increases the odds for Chagas disease transmission. Similarly, if socio‐economic conditions do not improve, the population at risk of Chagas disease transmission will continue to grow in Western Venezuela, despite the potential for ecosystem changes that could reduce the area of habitats suitable for *R. prolixus* optimal development, as expected by large scale land use and land cover transformation (Gottdenker et al., 2014). In that sense, long‐term robust interventions used to control Chagas disease include housing improvement (Berti et al., 1962; Briceño‐León, 1987; Monroy et al., 2009). However, housing improvement and many other potential solutions to reduce Chagas disease transmission, which could go coupled with poverty elimination and the solution to other social and economic problems shaping Chagas disease transmission, are at odds with current neoliberal policies that have repeatedly failed to reduce poverty and improve livelihoods in Latin America and elsewhere around the planet (Briceño‐León, 2007; Wallace et al., 2018). Neoliberal political economy is also one of the reasons behind the inability to reduce CO_2_ emissions and to address other causes of climate change (Ciplet & Roberts, 2017; Fremstad & Paul, 2022), and is also at the root of large ecosystem changes promoting disease transmission, such as the creation of agroecosystems that conflict with the promotion of a Structural One Health that effectively improves livelihoods (Gottdenker & Chaves, 2024; Perfecto et al., 2023; Wallace et al., 2015).

Although SDMs are effective tools to map species distributions, they have limitations. For example, unobserved environmental conditions and ecological processes may confound the model estimates if they have direct impact on the species and, at the same time, they are correlated with the observed environmental covariates (Mäkinen et al., 2022). To avoid spatial confounding and preserve data quality, we spatially constrained our study area based on the extent of the sampled occurrences avoiding extrapolation not substantiated by field observations. We also validated our model predictions with an independent data set not considered in model fitting, which is a methodological advance in testing the accuracy of SDMs for kissing bugs. Our validation showed that higher habitat suitability was more likely to predict the presence of *R. prolixus*, and we considered this a step that should be more systematically pursued when generating SDMs. We also want to note that these results could have been sensitive to the number of locations tested. For example, if one of the high suitability locations in this sample was negative for the presence of *R. prolixus* results would have been negative. This calls for a detailed design when sampling insect vectors for the validation of SDMs with prospective data. We think that key for this success was the stratified nature of the sampling in this validation data set, which was done along the elevational range of the Venezuelan Andes (Añez et al., 2021), and the consideration of gradients in covariates used to generate SDMs should be considered when designing the sampling of vectors for validation of SDMs. We also think that future work could look at the interaction of bug abundance (not just kissing bug habitat suitability) and infection to better refine population at risk of Chagas disease estimates, which could also benefit from considering measurements of social vulnerability that increase the risk of infection (Briceño‐León, 1990). Similarly further research is needed to understand the impacts of temporal mismatches between entomological samples and environmental variables considered in the ensemble SDM. Although our map, based on data from 2004 to 2012, was successful to predict observations in the validated data set (from 2012 to 2018), temporal mismatches that are coupled with drastic changes in the environment could potentially reduce the predictive ability of SDMs.

The most recent population at risk estimates for Chagas disease in Venezuela (country‐wide estimates) are from 2018, where PAHO estimated that 9,827,461 were exposed to the vector. These results can't be directly compared, due to differences in geographic extent, with our estimates. However, the methodology employed for the PAHO estimation is based on considering population estimates for areas where “documented presence of domiciliary vectors (allochthonous or autochthonous) and/or active vector transmission …” of Chagas disease as assessed by national health authorities (PAHO, 2025). We consider that our methodology allows for more precise estimates that can be scaled up for the generation of country‐wide estimates as programs for entomological surveillance develop. For example, high quality entomological data is collected for dengue vectors in Costa Rica as part of a methodologically homogeneous national program (Chaves et al., 2021), while in the USA local vector control agencies collect data that can also be used for producing high quality maps for vectors (Hamer, 2016). Similarly, on the side of population counts more precise estimates could be possible using other data sources for population estimates, such as those based on nighttime light data, which can provide very precise population estimates at a finely grained spatial resolution that is calibrated by nighttime light intensity across a landscape (McAvoy & Vadrevu, 2024). In this sense, considering that the finest‐grain demographic data for Venezuela is collected at the “parroquia” level, political‐administrative units historically based on population attendance at churches (Chaves, 1978, 1983; Siso Quintero, 2012), our estimates, for example, could average population counts by area (Lewontin & Levins, 1989), a problem also present in PAHO estimates, which, as we already expressed, are also likely to have imprecise estimates about the geographical extent of dominant Chagas disease vectors (PAHO, 2025).

## 6 Conclusions

Our results show the feasibility of generating ensemble SDMs using remotely sensed data. Our results also highlight the importance of considering higher order moments of environmental variables, for example, SD and kurtosis, when modeling species distribution as expected from Schmalhausen's law (Chaves, 2017). Our results suggest that *R. prolixus* is more likely to be present in low elevation areas with intermediate vegetation (also sparse, with platykurtic distributions), high temperatures with low variability (low SD and leptokurtic distributions). Finally, we illustrate how SDMs can be used to estimate population at risk of Chagas disease. Based on the SDM we estimated that as of 2020 between 82,296 and 1,943,934 people could be at risk of Chagas disease transmission in the study area of western Venezuela, based on the entomological risk of transmission that emerges from the overlap of vectors and human populations. In this study we focus on *R. prolixus* because it is the dominant vector species in our studied area, however, we are aware that there are other important kissing bug species able to transmit *T. cruzi* that also inhabit palms (Añez et al., 2017, 2021; Rodríguez et al., 2018), and whose distribution and role in *T. cruzi* transmission also needs to be studied. Similarly, we are aware that risk estimations could become more accurate if other factors important for transmission, such as housing quality and other social factors key for the successful transmission of *T. cruzi* parasites by *R. prolixus* (Briceño‐León, 1987; Feliciangeli et al., 2003) are considered. Also, recent developments in data science could even allow the development of data assimilation pipelines that enable near real time estimates of vector presence and/or abundance. Finally, we want to stress that these and other estimates can be very valuable when they are based on a nuanced knowledge of the wider socio‐ecological‐epidemiological contexts of transmission (Chaves et al., 2023, 2024; Salomon, 2025) which goes well beyond the sake of accurate predictions that don't advance actionable knowledge.

## Conflict of Interest

The authors declare no conflicts of interest relevant to this study.

## Erratum

The originally published version of this article omitted the award ID from the Acknowledgments and the funding section. The thrid sentence of the Acknowledgments has been corrected as follows: LFC was funded by NSF CNH2‐1924200. The second funder has been corrected as follows: National Science Foundation CNH2‐1924200. This may be considered the authoritative version of record.

## Data Availability

Geographical coordinates for occurrence points used for model fitting are available in Añez et al. (2017), while geographical coordinates for occurrences and absences used for model validation are available in Añez et al. (2021). These two data sets and the raster for the species distribution model presented in Figure 5, and the uncertainty in Figure 6, are available in Chaves (2026). All analyses were performed with the open access statistical software *R* and the open access geographical information science software QGIS using packages and algorithms described in the methods section of this article.
